# The Impact of Frailty on Postoperative Complications in Total En Bloc Spondylectomy for Spinal Tumors

**DOI:** 10.3390/jcm12124168

**Published:** 2023-06-20

**Authors:** Masafumi Kawai, Satoru Demura, Satoshi Kato, Noriaki Yokogawa, Takaki Shimizu, Yuki Kurokawa, Motoya Kobayashi, Yohei Yamada, Satoshi Nagatani, Takaaki Uto, Hideki Murakami

**Affiliations:** 1 Department of Orthopaedic Surgery, Graduate School of Medical Sciences, Kanazawa University, Kanazawa 920-8641, Japan; 2 Department of Orthopaedic Surgery, Graduate School of Medical Sciences, Nagoya City University, Nagoya 467-8601, Japan

**Keywords:** spinal tumors, total en bloc spondylectomy (TES), complications, risk factor, frailty, 5-factor Modified Frailty Index (mFI-5)

## Abstract

Total en bloc spondylectomy (TES) is an effective treatment for spinal tumors. However, its complication rate is high, and the corresponding risk factors remain unclear. This study aimed to clarify the risk factors for postoperative complications after TES, including the patient’s general condition, such as frailty and their levels of inflammatory biomarkers. We included 169 patients who underwent TES at our hospital from January 2011–December 2021. The complication group comprised patients who experienced postoperative complications that required additional intensive treatments. We analyzed the relationship between early complications and the following factors: age, sex, body mass index, type of tumor, location of tumor, American Society of Anesthesiologists score, physical status, frailty (categorized by the 5-factor Modified Frailty Index [mFI-5]), neutrophil-to-lymphocyte ratio, C-reactive protein/albumin ratio, preoperative chemotherapy, preoperative radiotherapy, surgical approach, and the number of resected vertebrae. Of the 169 patients, 86 (50.1%) were included in the complication group. Multivariate analysis showed that high mFI-5 scores (odds ratio [OR] = 2.99, *p* < 0.001) and an increased number of resected vertebrae (OR = 1.87, *p* = 0.018) were risk factors for postoperative complications. Frailty and the number of resected vertebrae were independent risk factors for postoperative complications after TES for spinal tumors.

## 1. Introduction

Total en bloc spondylectomy (TES) is an aggressive surgical treatment developed for the complete resection of spinal tumors, including primary malignant, aggressive benign, and metastatic tumors [1,2]. This procedure is an important option for reducing local tumor recurrence and improving patient prognosis [3,4,5,6,7,8]. Previous studies have reported favorable outcomes with regard to postoperative survival and local controls not only in cases of primary spinal tumors but also in those of solitary spinal metastases, particularly those originating from renal and thyroid cancers [9,10,11,12,13,14,15]. However, TES is a technically challenging procedure and carries the risk of injury to the spinal cord, major vessels, and organs, including the lungs, owing to these anatomical features’ proximity to the area being operated on. Moreover, the rate of postoperative complications such as infection and cardiovascular disorders, including deep vein thrombosis and pulmonary embolism, is high because of long operation times, large volumes of blood loss, and prolonged bed rest [8,16,17,18,19,20,21,22]. Despite the high rate of complications, there are only a few reports on perioperative complications pertaining to TES, and most are related to the surgical technique employed, such as the number of vertebrae resected or the use of the anterior–posterior approach, rather than the patient’s general condition [17,21,22].

Recently, it has been reported that “frailty”, an age-related clinical condition characterized by decreased physiological capacity and increased susceptibility to stressors, has been associated with perioperative complications and prognosis in various fields [23,24,25,26], including spine surgery [27,28,29]. Frailty is particularly important in relation to neoplasms, for which frail or pre-frail patients are at increased risk of suffering from perioperative complications, chemotherapy resistance, disease progression, and death [30,31,32,33]. However, no studies have examined the importance of frailty in radical spinal tumor surgery.

This study aimed to identify the risk factors for complications among patients who underwent TES for spinal tumors based on patient and surgical factors, including frailty.

## 2. Materials and Methods

### 2.1. Study Design

After obtaining approval from the Institutional Review Board of our university (IRB number: 2015-075), a database of patients who underwent TES for thoracic or lumbar spinal tumors in order to provide a locally curative treatment at our institution between January 2011 and December 2021 was retrospectively reviewed at the end of 2022. A total of 169 patients were included in this study, and no patients were excluded as we were able to collect complete data for all cases. At our institution, TES was indicated based on the following criteria: primary (malignant or aggressive benign) or solitary metastatic lesions, removable lesions (tumors involving ≤ 3 contiguous vertebrae), operability (Eastern Cooperative Oncology Group [ECOG] Performance Status ≤ 3), and stable disease with no or a limited number of metastases in non-spinal lesions. All metastases outside the surgical site were effectively managed using chemotherapy and radiotherapy. A detailed description of the surgical indications and significances of TES for metastatic lesions has been previously presented [34]. Informed consent for surgery was obtained from all patients. At our institute, pre- and postoperative radiotherapy was not employed for the patients who underwent TES. For patients with local tumor recurrence in the operated spine after TES, radiotherapy with or without additional surgery was considered. For all patients who underwent preoperative radiotherapy in this study, treatment was administered at another hospital before referral to our hospital for TES. Patients who experienced postoperative complications that required additional intensive treatments were categorized into the complication group. Complications were categorized as respiratory (pneumonia, pleural effusion, pneumothorax, and respiratory failure), operative-wound-related (surgical site infection [SSI] and wound dehiscence), neurological, cerebrospinal-fluid (CSF)-leakage-related, cardiovascular (heart failure, deep vein thrombosis, and pulmonary embolism), genitourinary (urinary retention and urinary tract infection), gastrointestinal (ileus and gastric ulcer), and others. Neurological complications were defined as a decrease in the Frankel grade of at least one level compared to the preoperative level. Complications were classified in terms of severity according to McDonnell et al. [35], with complications that would substantially alter the smooth and expected course of recovery considered major complications and others considered minor complications. Postoperative complications were defined as those that occurred within 2 months after surgery or during hospitalization; implant-related complications and late complications (>2 months after surgery) were excluded.

### 2.2. Data Collection

Patient information was obtained from the prospective study database. Missing information was obtained by adding it to medical records. Patient clinical data collected included age, gender, body mass index (BMI), comorbidities (hypertension requiring medication, diabetes, congestive heart failure, and chronic obstructive pulmonary disease), American Society of Anesthesiologists physical status (ASA-PS) classification, preoperative chemotherapy, preoperative radiotherapy, type of tumor (primary or metastatic or primary unknown), location of the tumor (thoracic spine [T1 to T12] and lumbar spine [L1 to L5]), surgical approach (posterior-only or anterior–posterior combined), the number of resected vertebrae, operative time, estimated blood loss, neutrophil-to-lymphocyte ratio (NLR), and C-reactive protein/albumin ratio (CAR).

### 2.3. Frailty Index

Frailty was categorized using the 5-factor Modified Frailty Index (mFI-5) [36]. The index was calculated based on the presence of five comorbidities: congestive heart failure within 30 days, diabetes mellitus, chronic obstructive pulmonary disease, partially or totally dependent functional health status, and hypertension requiring medication. We categorized patients with a score of 0 as cases of non-frailty, with a score of 1 as cases of pre-frailty, and with a score of 2 or more as cases of frailty.

### 2.4. Statistical Analysis

Categorical variables are described in terms of frequencies and percentages, continuous variables with a normal distribution are described in terms of mean and standard deviation (SD), and continuous variables with a non-normal distribution are described in terms of the median and interquartile range (25% and 75%). We performed Student’s *t*-test and Mann–Whitney U test for parametric and nonparametric data, respectively. The chi-square test was used to analyze categorical data, and Fisher’s exact test was used for smaller sample sizes. The Cochran–Armitage trend test was used to analyze the trends among the non-frailty, pre-frailty, and frailty groups. Multivariate stepwise logistic regression was used to adjust for potential confounders and identify independent risk factors for postoperative complications. Covariates associated with postoperative outcomes in univariate analyses were candidates for multiple logistic regression. All data were analyzed using IBM SPSS Statistics software version 27 (IBM, Armonk, NY, USA), with statistical significance set at *p* < 0.05.

## 3. Results

Table 1 shows the characteristics and surgical data of the patients with and without postoperative complications. The study cohort included 44 patients with primary tumors and 125 with metastatic tumors. Of the 44 patients with primary tumors, the most common pathology was a giant cell tumor, which was present in 12 patients, followed by aggressive hemangioma in 9, osteosarcoma in 6, and other pathologies in 17. Of the 125 patients with metastatic tumors, the primary site was the kidney in 46 patients, lungs in 16, breast in 13, thyroid in 9, sarcoma in 8, and other sites in 33. According to the ASA-PS classification, 4 patients corresponded to class I (2.4%), 143 patients corresponded to class II (84.6%), 22 patients corresponded to class III (13%), and none corresponded to class IV or more. Additionally, 97 (57%), 52 (31%), 15 (9%), and 5 (3%) of patients had mFI-5 scores of 0, 1, 2, and 3. No patients had mFI-5 scores higher than 4. Of the patients, 53 (31.4%) received preoperative chemotherapy, and 38 (22.5%) received preoperative radiotherapy. Surgical approaches were posterior in only 140 cases and anterior–posterior combined in 29 cases. A total of 129 patients (76.3%) had one vertebra resected, 18 (10.1%) had two vertebrae resected, and 22 (13.6%) had three vertebrae resected. In our study of 125 cases of spinal metastases, 54 (43.2%) exhibited metastases outside the surgical site. No association was found between the presence of metastases at other sites and the occurrence of complications (*p* = 0.136).

**Figure 1:**
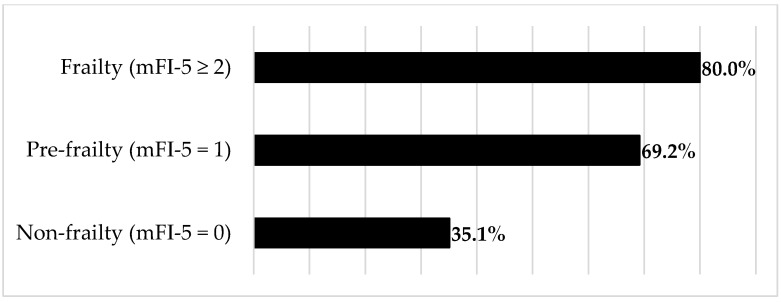
Complication rate per frailty. Cochran–Armitage trend test showed a significant difference (*p* < 0.001).

Of the 169 patients, 86 (50.1%) were assigned to the complication group, and the remaining 83 (49.9%) were assigned to the non-complication group. A total of 161 complications (72 major and 89 minor) occurred in 86 patients in the complication group (Table 2). Respiratory complications were the most common, followed by operative wounds, neurological complications, and CSF leakage. Significant differences were found between the complication and non-complication groups in terms of ASA-PS score, frailty, NLR, CAR, preoperative radiotherapy, number of resected vertebrae, and operative time (Table 1). In particular, the complication rate increased with increasing frailty and the number of resected vertebrae. The trend test showed a significant difference in the complication rate of frailty (*p* < 0.001): 35.1% for non-frailty (mFI-5 = 0), 69.2% for pre-frailty (mFI-5 = 1), and 80% for frailty (mFI-5 ≥ 2) (Figure 1). There was a trend towards a higher complication rate when more vertebrae were resected (44.1% for one vertebra, 72.2% for two vertebrae, and 72.7% for three vertebrae, with no significant difference according to the trend test). With regard to multiple logistic regression analysis, for which age, sex, and six items that showed significant differences in the univariate analysis were used as explanatory variables, high mFI-5 scores (odds ratio [OR] 2.99, 95% confidence interval [CI] 1.76–5.09; *p* < 0.001) and an increased number of resected vertebrae (OR 1.86, 95% CI 1.11–3.14; *p* = 0.018) were independent risk factors for postoperative complications. Additionally, in multivariate analysis with major complications serving as the dependent variable, high mFI-5 scores (OR 1.83, 95% CI 1.16–2.91; *p* = 0.010) and an increased number of resected vertebrae (OR 2.39, 95% CI 1.48–3.87; *p* < 0.001) were also independent risk factors (Table 3).

To present the characteristics of patients with frailty related to postoperative complications, we compared the clinical data of patients with and without pre-frailty/frailty. Patients with pre-frailty/frailty were older, had a more severe ASA-PS grade, and had higher NLR and CAR values than those without frailty. Male patients and patients with metastatic tumors were more common in the pre-frailty/frailty group (Table 4). The complication rates differed significantly between the non-frailty (35.1%) and pre-frailty/frailty (72.2%) groups. In the pre-frailty/frailty group, nearly half of the patients (48.6%) experienced major complications, which was significantly higher than the incidence of major complications in the non-frailty group. Neurological, cardiovascular, and gastrointestinal complications were comparable between the two groups. However, they were significantly more common in the respiratory, operative wound, CSF leakage, and genitourinary complications groups than in the non-frailty group.

## 4. Discussion

Although TES is an important treatment option for spinal tumors, it is technically difficult, highly invasive, and has a high complication rate. Several previous reports have concerned the complications of the en bloc resection of spinal tumors, mostly in small case series. Some large studies have reported complications in 41.7% to 67% of cases after en bloc resection [17,21,22,37]. A systematic review of 36 studies of 961 patients who underwent en bloc resection for spinal tumors, including several case reports, found complications in 58.3% of patients [38]. This is comparable to the complication rate of 50.1% observed in the present study. However, our study included only complications in the early postoperative period without implant failures because we aimed to identify the risk factors for early postoperative complications among patients who underwent TES for spinal tumors. This systematic review reported that neurological complications were the most common, with a rate of 12.7%, which is consistent with the rate of 14.8% observed in this study. In our study, respiratory and wound-related complications were more common than neurological complications. This may be due to differences in the classification of complications. We included pleural effusions requiring drainage or thoracentesis as a respiratory complication, but this complication was not included in the large studies reported by Boriani [17] and Kurokawa et al. [39] presented in their reviews. Respiratory complications were identified as the most common in the study by Demura et al., who classified pleural effusions as respiratory complications [21]. They discussed direct surgical invasion, especially with an accompanying circumferential dissection around the thoracic vertebrae for en bloc resection, which may result in massive pleural effusion. Similarly, the rate of wound-related complications was elevated in our study owing to the inclusion of wound dehiscence and SSI.

In several reports, surgical approach, previous surgery, previous treatment (neoadjuvant chemotherapy and/or radiotherapy), age > 50 years, and the number of resected vertebrae have been reported to be risk factors for complications with respect to the en bloc resection of spinal tumors [17,21,22,37]. Amendola et al. reported on the en bloc resection of primary spinal tumors in 103 patients and found increased complications with an anterior–posterior combined approach compared to a single posterior approach. Another study comparing anterior and posterior approaches for thoracic spinal metastases showed a significant difference, with complications occurring in 6 of 25 patients (24%) in the anterior group and 6 of 72 patients (8.3%) in the posterior group [40]. Complication rates were higher with the anterior–posterior approach in our study, although the difference was not statistically significant. In addition to an anterior–posterior combined approach, Bandiera and Boriani et al. reported that neoadjuvant chemotherapy and radiotherapy were independent risk factors for complications after en bloc resection [17,38]. In our study, although it was not a significant factor in the multivariate analysis, the association with a history of preoperative radiotherapy was higher in the complication group than in the non-complication group. Several previous studies showed that preoperative conventional radiotherapy increased perioperative complications, especially wound complications [41,42,43]. Additionally, dural damage has been reported to occur due to spinal epidural fibrosis and the thinning of the arachnoid barrier cell layer after irradiation [44]. In a previous study, it was reported that preoperative radiotherapy increased wound complications and CSF leakage after TES [45]. In contrast, neoadjuvant chemotherapy has not been associated with increased postoperative complications [46,47,48].

This study identified the number of resected vertebrae and frailty as risk factors for postoperative complications associated with TES. In a large, single-center study, Demura et al. reported that the number of resected vertebrae was a risk factor for perioperative complications, including late complications [21]. They showed a significant increase in hardware failure and respiratory and cardiovascular complications in a group with more than two vertebrae resected. In our study, which also analyzed the risk of early postoperative complications, including patient factors such as frailty and inflammatory biomarkers, the number of resected vertebrae was an independent predictor of complications, with a 1.9-fold increase in complications for each additional resected vertebra.

Previous reports have focused on technical issues and the local environment, such as tumor progression and radiotherapy. In contrast, this study is the first to report the influence of a patient’s general condition, such as frailty, inflammatory biomarkers, on TES complications. Recently, frailty has been reported to be superior to age in terms of being a predictor of mortality, complications, and length of stay [23,24,49,50,51,52]. Several studies on spinal surgery, particularly for spinal metastasis, have suggested that frailty is an important predictor of outcomes [29,53]. The mFI-5 is reported to be a clinically useful frailty scale that is easy to calculate and incorporates objectivity, reproducibility, and generalizability [53]. The mFI-5 was also a useful predictor of complications in the present study. Bakhsheshian et al. reported that frailty is a useful predictor of greater medical complications, surgical complications, nonroutine discharges, and costs among patients who undergo surgery for spinal metastasis [54]. Of the 7772 patients, 1974 (25.4%) were frail, with a higher prevalence of frailty among patients who had undergone surgery for spinal tumors. Our study found that frailty was an independent predictor of complications after TES and that increasing frailty was associated with more respiratory, wound-related, and genitourinary complications. Pulmonary function was lower in frail and prefrail adults than in non-frail adults [55]. The risk of perioperative respiratory complications may increase due to decreased lung reserves. Nutritional disorders suffered by frail patients may also depress their neural drive and muscular strength, both of which may contribute to reduced diaphragmatic function and respiratory complications after the procedure [56]. Similarly, postoperative infections, such as SSI or urinary tract infections, are preceded by frailty [57,58]. In general, undernutrition among frail patients impedes the processes involved in postoperative recovery [59,60]. Protein energy undernutrition impairs wound healing and immunological defenses, thus elevating the risk of infection [61]. Patients undergoing TES are often younger and in better general condition than those undergoing palliative surgery because patients referred to specialized institutions, such as our institutions, are considered ideal candidates for aggressive spinal tumor surgery and are in a relatively stable general condition. Therefore, there is a possibility that commonly used indices that comprehensively evaluate the preoperative overall health status of patients, such as ASA-PS, ECOG-PS, and the Karnofsky Performance Scale, may not adequately stratify patients. However, frail TES patients do exist.

Notably, even pre-frail conditions are associated with higher complication rates. Young and relatively healthy patients can be classified as pre-frail if they have comorbidities such as diabetes or hypertension, indicating the need to assess their frailty status and be aware of complications. Surgeons must remember that alternative treatment options, such as radiotherapy or palliative surgery, should be considered for patients with advanced frailty, particularly in cases of metastatic tumors.

The present study has several limitations, including its retrospective design, relatively small cohort size in a single center, and heterogeneity in tumor type. It included various types of primary and metastatic tumors, and the type of tumor might have affected the analyzed patients’ general condition, the amount of intraoperative blood loss, and the difficulty of the operation. In addition, we could not obtain detailed information including the indication of radiation and systemic therapy because these therapies, executed by primary physicians, were mostly performed at other hospitals. In addition, there might have been selection bias, as patients referred to the department are often relatively good candidates for TES. Despite these limitations, this study indicates that frailty is a useful predictor of postoperative complications and helps surgeons select patients for TES.

## 5. Conclusions

Half of the patients who underwent TES for spinal tumors experienced early postoperative complications. Frailty and the number of resected vertebrae were independent risk factors associated with postoperative complications after TES. It is important to perform a preoperative assessment of a patient’s comprehensive frailty status and manage the risk of complications.

## Figures and Tables

**Table 1:** Characteristics and surgical data of patients with and without postoperative complications.

Variable	Total (*n* = 169)	Non-Complications (*n* = 83)	Complications (*n* = 86)	*p*-Value
Age (years), mean ± SD	53.3 (14.3)	55.0 (14.1)	51.5 (14.4)	0.113
Sex				0.079
- Male, *n* (%)	99 (58.6)	43 (51.8)	56 (65.1)	
- Female, *n* (%)	70 (41.4)	40 (48.2)	30 (34.9)	
BMI (mean ± SD)	22.4 (3.6)	21.9 (3.5)	22.8 (3.7)	0.137
Type of tumors, *n* (%)				0.124
- Primary	44 (26.0)	26 (31.3)	18 (20.9)	
- Metastatic	125 (74.0)	57 (68.7)	68 (79.1)	
Location of tumors, *n* (%)				0.41
- Thoracic spine (T1 to T12)	119 (70.4)	56 (67.5)	63 (73.3)	
- Lumbar spine (L1 to L5)	50 (29.6)	27 (32.5)	23 (26.7)	
ASA-PS, *n* (%)				0.005 *
- I	4 (2.4)	3 (3.6)	1 (1.1)	
- II	43 (84.6)	75 (90.4)	68 (79.1)	
- III	22 (13.0)	5 (6.0)	17 (19.8)	
mFI-5, *n* (%)				<0.001 *
- 0	97 (57.4)	63 (75.9)	34 (39.5)	
- 1	52 (30.8)	16 (19.3)	36 (41.9)	
- 2	15 (8.9)	4 (4.8)	11 (12.8)	
- 3	5 (2.9)	0	5 (5.8)	
NLR, median (IQR)	2.64 (1.87, 3.95)	2.22 (1.70, 3.34)	3.21 (1.93, 4.61)	0.015 *
CAR, median (IQR)	0.027 (0.010, 0.110)	0.024 (0.002, 0.050)	0.048 (0.004, 0.163)	<0.001 *
Preoperative chemotherapy, *n* (%)	53 (31.4)	28 (33.7)	25 (29.1)	0.513
Preoperative radiotherapy, *n* (%)	38 (22.5)	12 (14.5)	26 (30.2)	0.014
Surgical approach, *n* (%)				0.36
- Posterior only	140 (82.8)	71 (85.5)	69 (80.2)	
- Anterior–posterior combined	29 (17.2)	12 (14.5)	17 (19.8)	
The number of resected vertebrae, *n* (%)				0.007 *
- 1	129 (76.3)	72 (86.7)	57 (66.3)	
- 2	18 (10.1)	5 (6.0)	13 (15.1)	
- 3	22 (13.6)	6 (7.3)	16 (18.6)	
Operative time (min), median (IQR)	447 (360, 559)	399 (338, 535)	476 (396, 567)	0.002 *
Estimated blood loss (mL), median (IQR)	420 (235, 590)	400 (200, 600)	430 (265, 585)	0.338

SD, standard deviation; BMI, body mass index; ASA-PS, American Society of Anesthesiologists physical status; mFI-5, 5-factor Modified Frailty Index; NLR, neutrophil/lymphocyte ratio; IQR, interquartile range; CAR, C-reactive protein/albumin ratio; * *p* < 0.05.

**Table 2:** Overall postoperative complications.

Type of Complications	Total	Major	Minor
Total, *n* (%)	161	72 (44.7)	89 (55.3)
Respiratory	37 (41.4)	12 (16.2)	25 (27.8)
Operative wound	28 (17.3)	13 (17.6)	15 (16.7)
Neurological	25 (15.4)	12 (16.2)	13 (14.4)
CSF leakage	24 (14.8)	11 (14.9)	13 (14.4)
Cardiovascular	16 (9.9)	7 (9.5)	9 (10.0)
Genitourinary	11 (6.8)	1 (1.4)	10 (11.1)
Gastrointestinal	7 (4.3)	5 (6.8)	2 (2.2)
Others	13 (8.0)	11 (14.9)	2 (2.2)

CSF, cerebrospinal fluid.

**Table 3:** Multivariate analysis of factors associated with postoperative complications after TES.

	OR	*p*-Value	95% CI
Total complications			
- mFI-5 score	2.99	<0.001	1.76–5.09
- number of resected vertebrae	1.87	0.018	1.11–3.14
Major complications			
- mFI-5 score	1.83	0.010	1.16–2.91
- number of resected vertebrae	2.39	<0.001	1.48–3.87

TES, total en bloc spondylectomy; OR, odds ratio; CI, confidence interval; mFI-5, 5-factor Modified Frailty Index.

**Table 4:** Comparison between the non-frailty and pre-frailty/frailty groups.

Variable	Non-Frailty (mFI-5 = 0, *n* = 97)	Pre-Frailty/Frailty (mFI-5 ≥ 1, *n* = 72)	*p*-Value
Age (years), mean ± SD	47.9 (14.4)	60.6 (10.6)	<0.001 *
Sex			<0.001 *
- Male, *n* (%)	44 (45.4)	55 (76.4)	
- Female, *n* (%)	53 (54.6)	17 (23.6)	
BMI, mean ± SD	22.0 (3.8)	22.8 (3.3)	0.143
Type of tumors, *n* (%)			<0.001 *
- Primary	35 (36.1)	9 (12.5)	
- Metastatic	62 (63.9)	63 (87.5)	
Location of the tumor, *n* (%)			0.071
- Thoracic spine (T1 to T12)	63 (64.9)	56 (77.8)	
- Lumbar spine (L1 to L5)	34 (35.1)	16 (22.2)	
ASA-PS, *n* (%)			<0.001 *
- I	4 (4.1)	0	
- II	89 (91.8)	54 (75.0)	
- III	4 (4.1)	18 (25.0)	
NLR, median (IQR)	2.25 (1.72, 3.43)	3.36 (2.06, 4.88)	0.002 *
CAR, median (IQR)	0.024 (0.004, 0.527)	0.049 (0.022, 0.250)	0.001 *
Preoperative chemotherapy, *n* (%)	30 (30.1)	23 (31.9)	0.888
Preoperative radiotherapy, *n* (%)	17 (17.5)	21 (29.2)	0.073
Surgical approach, *n* (%)			0.166
- Posterior only	77 (79.4)	63 (87.5)	
- Anterior–posterior combined	20 (20.6)	9 (12.5)	
The number of resected vertebrae, *n* (%)			0.227
- 1	78 (80.4)	51 (70.8)	
- 2	10 (10.3)	8 (11.1)	
- 3	9 (9.3)	13 (18.1)	
Total operative time (min), median (IQR)	455 (355, 576)	445 (359, 547)	0.724
Estimated blood loss (mL), median (IQR)	400 (215, 600)	430 (240, 570)	0.675
Complications, *n* (%)			
- Total	34 (35.1)	52 (72.2)	<0.001 *
- Major	20 (20.6)	35 (48.6)	<0.001 *
- Respiratory	11 (11.3)	22 (30.6)	0.002 *
- Operative wound	8 (8.2)	19 (26.4)	0.001 *
- Neurological	12 (12.4)	13 (18.1)	0.303
- CSF leakage	6 (6.2)	18 (25.0)	<0.001 *
- Cardiovascular	6 (6.2)	8 (11.1)	0.251
- Genitourinary	1 (1.0)	10 (13.9)	<0.01 *
- Gastrointestinal	2 (2.1)	5 (6.9)	0.119
- Others	2 (2.1)	8 (11.1)	0.016 *

mFI-5, 5-Factor Modified Frailty Index; SD, standard deviation; BMI, body mass index; ASA-PS, American Society of Anesthesiologists physical status; NLR, neutrophil-to-lymphocyte ratio; IQR, interquartile range; CAR, C-reactive protein/albumin ratio; * *p* < 0.05.

## Data Availability

The datasets used and analyzed in the current study are available from the corresponding author upon reasonable request. The data are not publicly available because of privacy and ethical restrictions.
